# Increase in blood–brain barrier leakage in healthy, older adults

**DOI:** 10.1007/s11357-020-00211-2

**Published:** 2020-06-29

**Authors:** Inge C. M. Verheggen, Joost J. A. de Jong, Martin P. J. van Boxtel, Ed H. B. M. Gronenschild, Walter M. Palm, Alida A. Postma, Jacobus F. A. Jansen, Frans R. J. Verhey, Walter H. Backes

**Affiliations:** 1 Department of Psychiatry and Neuropsychology, Maastricht University, P.O. Box 616, 6200 MD Maastricht, The Netherlands; 2 School for Mental Health and Neuroscience (MHeNs), Maastricht University, P.O. Box 616, 6200 MD Maastricht, The Netherlands; 3 Alzheimer Center Limburg, P.O. Box 616, 6200 MD Maastricht, The Netherlands; 4 Department of Radiology and Nuclear Medicine, Maastricht University Medical Center, Maastricht, The Netherlands; 5 Department of Electrical Engineering, Eindhoven University of Technology, Eindhoven, The Netherlands; 6 Cardiovascular Research Institute Maastricht (CARIM), Maastricht University, Maastricht, The Netherlands

**Keywords:** Blood–brain barrier, Normal aging, Dynamic contrast-enhanced MRI, Permeability

## Abstract

Blood–brain barrier (BBB) breakdown can disrupt nutrient supply and waste removal, which affects neuronal functioning. Currently, dynamic contrast-enhanced (DCE) MRI is the preferred in-vivo method to quantify BBB leakage. Dedicated DCE MRI studies in normal aging individuals are lacking, which could hamper value estimation and interpretation of leakage rate in pathological conditions. Therefore, we applied DCE MRI to investigate the association between BBB disruption and age in a healthy sample. Fifty-seven cognitively and neurologically healthy, middle-aged to older participants (mean age: 66 years, range: 47–91 years) underwent MRI, including DCE MRI with intravenous injection of a gadolinium-based contrast agent. Pharmacokinetic modeling was applied to contrast concentration time-curves to estimate BBB leakage rate in each voxel. Subsequently, leakage rate was calculated in the white and gray matter, and primary (basic sensory and motor functions), secondary (association areas), and tertiary (higher-order cognition) brain regions. A difference in vulnerability to deterioration was expected between these regions, with especially tertiary regions being affected by age. Higher BBB leakage rate was significantly associated with older age in the white and gray matter, and also in tertiary, but not in primary or secondary brain regions. Even in healthy individuals, BBB disruption was stronger in older persons, which suggests BBB disruption is a normal physiologically aging phenomenon. Age-related increase in BBB disruption occurred especially in brain regions most vulnerable to age-related deterioration, which may indicate that BBB disruption is an underlying mechanism of normal age-related decline.

Netherlands Trial Register number: NL6358, date of registration: 2017-03-24.

## Introduction

Currently, an increasing number of people are reaching older ages (Lutz et al. 2008), and studies into the causes and prevention of age-related disorders are becoming increasingly important. However, people who age without any overt pathological condition may still experience some degree of age-related decline (Woodruff-Pak 1997). This decline shows large interindividual differences (Rapp and Amaral 1992), while it remains unclear what factors determine whether someone will be strongly affected by age or hardly experience any age-related setback. Cerebral microvascular alterations, occurring over time and contributing to blood–brain barrier (BBB) breakdown, could be a promising topic to study in relation to normal age-related decline (Farkas and Luiten 2001; Norton et al. 2019).

In the cerebral microvasculature, the vessel wall of the small capillaries contains endothelial cells connected by tight junctions, forming the basis of the BBB (Zlokovic 2011). The BBB separates the blood from the brain parenchyma and regulates the delivery of energy metabolites and nutrients to the neurons, while preventing neurotoxins from entering the brain tissue. The endothelium contains specialized transport systems that allow nutrients to move from the blood to the brain, while waste products are removed in the opposite direction (Montagne et al. 2017; Zhao et al. 2015). The BBB thereby protects the brain from concentration fluctuations that occur in the blood stream, which is essential for neuronal and synaptic functioning (Zlokovic 2008).

Endothelial dysfunction, such as impaired endothelial cell proliferation and migration, is one of the cerebral microvascular alterations occurring over time, as studies have found more endothelial dysfunction in elderly individuals (Murugesan et al. 2012; Ungvari et al. 2018; Ungvari et al. 2013). Also, aging has been associated with nicotinamide adenine dinucleotide (NAD^+^) deficiency (Csiszar et al. 2019; Gomes et al. 2013; Yoshino et al. 2018). NAD^+^ is a coenzyme, enzyme precursor, or substrate involved in cellular proliferation and function, and the regulation of cellular energetics and mitochondrial metabolism (Bonkowski and Sinclair 2016; Gomes et al. 2013). Age-related NAD^+^ depletion has been associated with endothelial dysfunction, and restoring the NAD^+^ levels appears to protect the integrity of the cerebral microvasculature (Csiszar et al. 2019; Kiss et al. 2019; Mills et al. 2016; Tarantini et al. 2019; Zhang et al. 2016). Thus, NAD^+^ deficiency affecting endothelial integrity could be an important cerebral microvascular alteration that contributes to BBB disruption over time.

BBB disruption impairs the oxygen and glucose supply, which may produce hypoxia and hypoxia induced inflammation (Fernando et al. 2006; Raja et al. 2018). Subsequent pathological processes make the brain vulnerable to neuronal dysfunction and could even lead to neurodegeneration (Levit et al. 2020). Moreover, BBB disruption reduces the clearance of interstitial solutes from the brain and could result in accumulation of toxic waste products, such as amyloid-β (Aβ) in case of Alzheimer’s disease (AD) (Burgmans et al. 2013). Aβ was found to further impair neurovascular functioning and the cerebral circulation, even before the actual formation of plaques, suggesting that these neurovascular alterations are an early event in the pathological cascade (DiBattista et al. 2020; Iadecola et al. 1999; Niwa et al. 2002a; Niwa et al. 2002b). Imaging studies also support that neurovascular dysfunction and BBB disruption occur early in the development of AD (Iturria-Medina et al. 2016; Montagne et al. 2015; Montagne et al. 2016; van de Haar et al. 2016a).

A large meta-analysis has suggested that BBB permeability increases already as part of normal aging (Farrall and Wardlaw 2009). However, in these studies the blood/CSF albumin ratio was used as biomarker for BBB leakage, which does not differentiate between blood–brain and blood–cerebrospinal fluid leakage and also cannot localize leakage. Nowadays, a more direct method to detect subtle permeability values is dynamic contrast-enhanced (DCE) MRI (Raja et al. 2018). A gadolinium-based contrast agent is intravenously injected during scanning, to quantify and localize the spread of contrast from the blood plasma to the brain interstitial fluid, which is dependent on the extent of BBB disruption. To distinguish the rapidly circulating component from the slowly extravasating component, dual-time resolution can ideally be applied (Jelescu et al. 2011; van de Haar et al. 2017). This acquisition technique applies a fast sequence to capture steep signal changes during the initial circulation phase, and a slow sequence during the later leakage phase. Using this technique, significantly larger BBB leakage rate was found in persons with mild cognitive impairment (Wang et al. 2006), often considered the transition state to AD, and cerebral small vessel disease (Zhang et al. 2017), the leading cause of vascular dementia (Pantoni 2010). A recent study even demonstrated that older adults with early cognitive dysfunction had significantly more BBB leakage than individuals showing no cognitive impairment, even in the absence of any neurological or psychiatric condition (Nation et al. 2019).

These studies suggest that BBB breakdown is part of the normal aging process and occurs even in the absence of neurological disorders. However, DCE MRI studies investigating whether older individuals have stronger BBB disruption, even when these individuals are considered healthy, have not been conducted. The lack of DCE MRI studies in normal aging individuals could hamper the value estimation and interpretation of BBB leakage observed in recent studies on brain pathology. Therefore, we aimed to investigate the association between BBB leakage and age in healthy, middle-aged to older individuals using DCE MRI.

Higher-order brain regions involved in high-level cognition, such as decision making, planning and related executive functions, are the first brain regions to show age-related deterioration (Andrews-Hanna et al. 2007). After the higher-order brain regions, the association areas are most vulnerable to age-related impairment, while areas involved in primary sensory and motor functions tend to remain robust as a function of normal aging (Casey et al. 2005). Our first hypothesis was that older age would be associated with higher BBB leakage rate throughout the cerebral white and gray matter. Secondly, with BBB disruption possibly being an underlying mechanism for normal age-related deterioration, we hypothesized that the association between BBB leakage and age will be most clearly present in those regions most affected by aging, which are the higher-order brain regions.

## Methods

### Participants

Fifty-seven participants (mean age: 66 years, age range: 47–91 years) were recruited from participants of the Maastricht Aging Study (MAAS) (Jolles et al. 1995), in which 1823 people were cognitively followed from 1992 until 2005. Only normal aging individuals, considered healthy, were eligible for participation. In this study, “healthy” was defined as no substantial decrease in global cognition (Mini-Mental State Examination (MMSE; Folstein et al. 1975) score ≥ 25), no cognitive impairment due to substance abuse, and no great difficulties in performing activities of daily living (Disabilities Assessment of Dementia (DAD; Gélinas et al. 1999) score ≥ 90%), as well as no major neurological conditions (no diagnosis of dementia, prodromal dementia, mild cognitive impairment or any other psychiatric or neurological disorder, and no major structural brain abnormalities, brain surgery, or brain trauma as known from the medical history). Furthermore, individuals could only be included when they had no contraindications for MRI or use of gadolinium-based contrast agent (sufficiently functioning kidneys as indicated by an estimated Glomerular Filtration Rate (eGFR) > 30 mL/min).

### Sample characteristics

For each participant, age and sex were registered. Experienced neuroradiologists visually rated white matter lesion load according to the Fazekas scale (Fazekas et al. 1993) (W.P.) and brain atrophy using the global cortical atrophy (GCA) scale (Pasquier et al. 1996) (A.P.), to confirm our sample could be considered neurologically healthy.

Blood pressure was measured twice, and the mean systolic blood pressure was calculated. Weight and height were measured to calculate the body mass index (BMI). Participants were also asked about the occurrence of diabetes and their smoking habits, after which they were classified as diabetic or non-diabetic and smoker or non-smoker. Systolic blood pressure, BMI, diabetes, and smoking habits were taken into consideration as potential confounders, as these factors may influence neurovascular health and BBB integrity (Gustafson et al. 2007; Mazzone et al. 2010; Rapoport 1976; Starr et al. 2003).

### MRI acquisition

Sagittally oriented slices were acquired on a 3-tesla MRI scanner (Achieva TX, Philips Healthcare, Best, The Netherlands) with a 32-channel head coil. The imaging protocol consisted of 3D T1-weighted inversion recovery fast gradient echo (repetition time (TR)/inversion time (TI)/echo time (TE): 8/800/4 ms; flip angle: 8°; cubic voxel size: 1 mm) for anatomical reference, 3D T2-weighted fluid attenuation inversion recovery (FLAIR) (TR/TI/TE: 4800/1650/290 ms; flip angle: 90°; cubic voxel size: 1 mm, no slice gap) for the white matter hyperintensity (WMH) volume, and dual-time resolution dynamic contrast-enhanced (DCE) MRI for the leakage measurement.

Dual-time DCE MRI consisted of two alternating sequences with a saturation prepulse to provide two time resolutions: a fast sequence with a dynamic scan interval of 3.2 s for high temporal resolution to capture the steep signal changes during the initial circulation phase of the contrast agent and a slow sequence with a dynamic scan interval of 30.5 s with lower temporal but higher spatial resolution during the slower leakage phase of the contrast agent (van de Haar et al. 2017). The fast sequence consisted of 29 volumes (TR/TE/delay time (TD): 5.3/2.5/120 ms; voxel size: 2 × 2 × 5 mm), and the slow sequence consisted of 30 volumes (TR/TE/TD: 5.3/2.5/120 ms; voxel size: 1 × 1 × 2 mm). Before contrast agent administration, scans of both sequences were acquired. Next, during the fast sequence, gadolinium-based contrast agent (0.1 mmol/kg gadobutrol, Gadavist®, Bayer AG, Leverkusen, Germany) was intravenously injected in the antecubital vein (injection rate 3 mL/s, 20 mL saline flush). Beforehand, T1-mapping was performed to enable conversion of tissue signal intensity to contrast agent concentration (Larsson et al. 2009).

### Brain segmentation

WMH volume (cm^3^) was determined by creating WMH masks on the FLAIR images using the in-house developed, semi-automatic segmentation tool GIANT (Jacobs et al. 2014). WMH volume was corrected for intracranial volume.

**Fig. 1:**
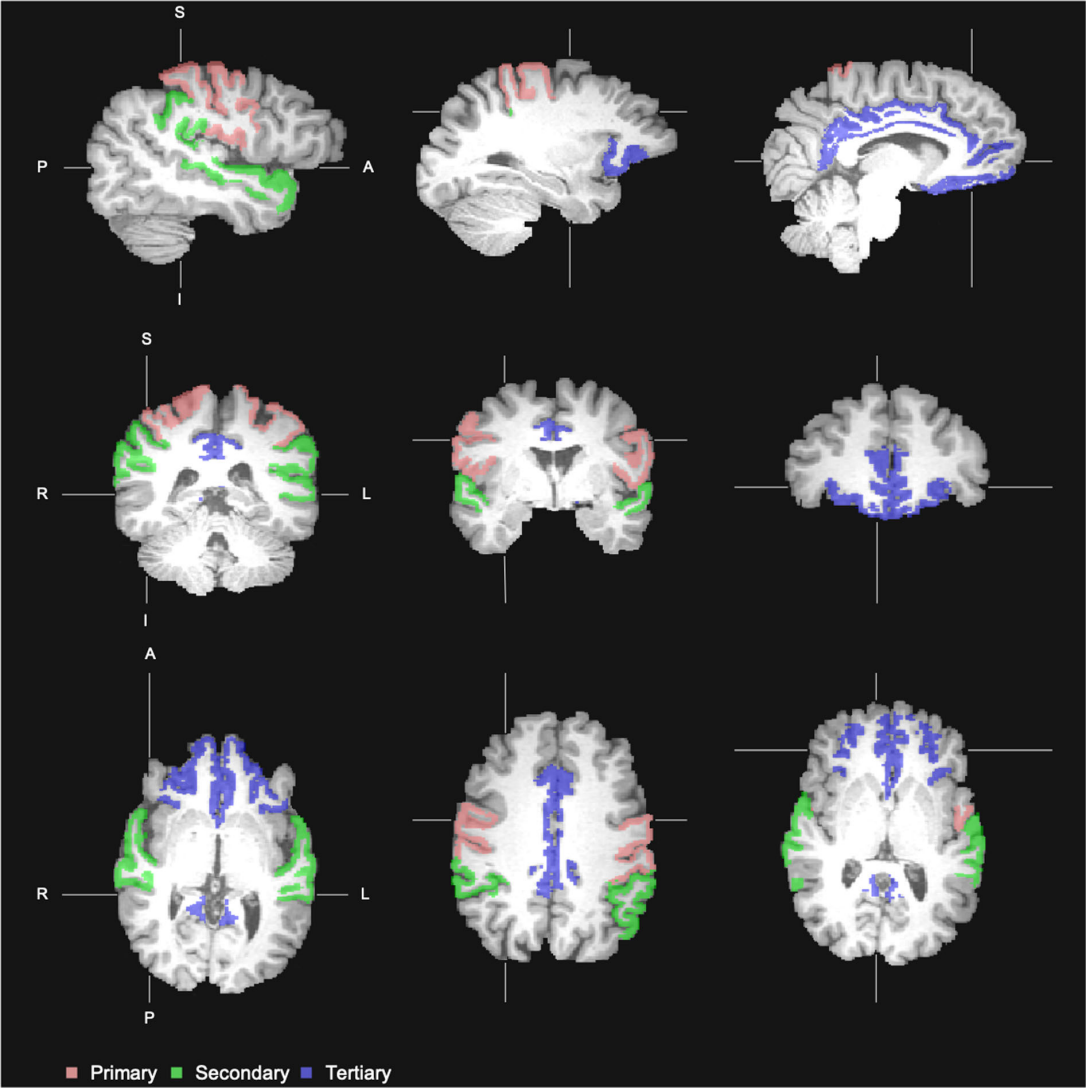
Cortical gray matter masks for the cortical regions of interest, with in pink: primary brain regions (precentral and postcentral gyrus), green: secondary brain regions (supramarginal and superior temporal gyrus), and blue: tertiary brain regions (orbitofrontal and cingulate cortex)

FreeSurfer software (version 6.0.0) was used for automated brain region segmentation (Fischl et al. 1999), combined with a visual check with manual adjustments (I.C.M.V.). From these segmentations, cortical thickness (mm) and hippocampal volume (cm^3^) were obtained. We refer to the brain regions involved in primary sensory and motor functions as primary brain regions, to the association areas as secondary brain regions and to the regions for higher-order cognitive functions as tertiary brain regions. The FreeSurfer software subdivides the cerebral cortex according to the Desikan–Killiany atlas (Desikan et al. 2006). Using this atlas, two brain regions were selected for each of the three classes: for the primary brain regions the precentral and postcentral gyrus, for the secondary brain regions the supramarginal and superior temporal gyrus, and for the tertiary brain regions the orbitofrontal and cingulate cortex (Fig. 1) (Andrews-Hanna et al. 2007; Casey et al. 2005). For our analyses, total white matter, total gray matter (cortical and deep gray matter and hippocampus), and primary, secondary, and tertiary brain regions were extracted to create tissue masks for each region of interest (ROI) (Fischl et al. 1999).

### DCE MRI processing

For both the fast and slow DCE MRI, a reference image was created using the average of the pre-contrast images in each sequence. The slow DCE MRI data were motion corrected, and the fast DCE MRI data were motion-corrected and co-registered to the slow DCE MRI data using a single transformation. The reference slow DCE MRI was registered on the T1-weighted anatomic reference images, and the inverse of the obtained transformation matrix was used to transform the T1-weighted data, including the primary, secondary, and tertiary brain regions, to slow DCE space.

At least 20 voxels in the superior sagittal sinus were manually selected (I.C.M.V.) to obtain a vascular input function (VIF) for each participant (Jelescu et al. 2011; van de Haar et al. 2017). Signal-to-concentration conversion was applied to the VIF using in vitro data (diluted MnCl_2_ stock solution with varying gadobutrol concentrations ranging from 1 to 40 mM and baseline T1 relaxation time comparable to human blood) and in the brain tissue by assuming a linear relationship and using the tissue relaxation time from the T_10_-map (van de Haar et al. 2016b).

### Pharmacokinetic model analyses

Using the participant-specific VIF, the Patlak approach (Patlak et al. 1983) was used for voxel-wise pharmacokinetic modeling of the contrast agent concentration in brain tissue and blood plasma. The Patlak model was found to be a suitable model for brain tissue and is the most parsimonious, as it assumes no reflux from the brain tissue back to the blood (Cramer and Larsson 2014).

With this approach, an estimation of the *K*_*i*_-parameter (min^−1^) was obtained for each voxel, which gives an indication of the leakage rate from the blood plasma to the brain tissue as a measure of permeability. Histograms of the *K*_*i*_ values in the white and gray matter and primary, secondary, and tertiary brain regions were calculated and corrected for noise (van de Haar et al. 2016a), after which the mean *K*_*i*_ was calculated for each region.

### Statistics

The mean *K*_*i*_ values appeared non-normally distributed and were therefore cube root-transformed to obtain a normal distribution. Multiple linear regression was performed with the mean *K*_*i*_ in each ROI as dependent variable and age and sex as predictors to investigate the association between BBB leakage and age while controlling for sex differences. Potential confounders, namely, mean systolic blood pressure, BMI, diabetes, and smoking habits, were also included in subsequent analyses to check whether they altered the results.

As additional post-hoc analyses, the influence of other measures of structural brain integrity, namely, WMH volume, cortical thickness, and hippocampal volume, was investigated. WMH volume values were log-transformed to obtain a normal distribution. Multiple linear regression was performed with *K*_*i*_ in each ROI as dependent variable, while age, sex, one of the brain integrity measures, and the interaction term between this measure and age were included as predictors. A non-significant interaction term was removed to test the significance of main effects.

All statistical analyses used a significance level of *p* < .05 and were performed with commercial software (SPSS, version 24.0, IBM Corp., Armonk, NY, USA).

## Results

The participant characteristics are presented in Table 1, showing high MMSE scores and low Fazekas and GCA ratings.

**Table 1:** Participant (*n* = 57) characteristics

	Mean (standard deviation)/percentage/median (25th–75th percentiles)	
Age	65.8 (10.2)	
% Male	52.6	
% Level of education^a^	1/2/3	15.8/54.4/29.8
MMSE^b^	29.0 (28.0–30.0)	
% WMH Fazekas^c^	0/1/2/3	5.3/70.2/12.3/12.3
% GCA^d^	0/1/2/3	19.3/50.9/24.6/5.3
Systolic blood pressure^e^	141.3 (17.2)	
% Diabetes	17.5	
BMI	27.7 (4.5)	
% Smoker	14.0	
eGFR	69.5 (12.6)	

^a^Level of education: 1 = at most primary or lower vocational education; 2 = secondary education; 3 = higher vocational or scientific education

^b^Mini-Mental State Examination (Folstein et al. 1975): maximum score = 30, cognitively normal ≥ 25

^c^Fazekas scale with a visual rating score of white matter hyperintensity load (Fazekas et al. 1993): 0 = absent: none or a single punctuate WMH lesion; 1 = mild: multiple punctuate lesions; 2 = moderate: beginning of confluency of lesions; 3 = severe: large confluent lesions

^d^Global cortical atrophy visual rating scale (Pasquier et al. 1996): 0 = absent: normal volume/no ventricular enlargement; 1 = mild: opening of sulci/mild ventricular enlargement; 2 = moderate: volume loss of gyri/moderate ventricular enlargement; 3 = severe: ‘knife blade’ atrophy/severe ventricular enlargement

^e^High blood pressure > 140 mmHg

For each ROI, the median and 25th–75th percentiles of the leakage rate and its association with age are presented in Table 2.

**Table 2:** Median and 25th–75th percentiles of the leakage rate in the regions of interest, including the standardized regression coefficient with age

ROI	Median leakage rate (× 10^−7^ min^−1^)	25–75 percentiles (× 10^−7^ min^−1^)	*β*^a^
White matter	11.4	5.1–20.4	.306*
Gray matter	9.5	3.9–17.3	.286*
Primary regions	4.4	1.0–10.8	.145
Secondary regions	3.4	0.4–7.1	.098
Tertiary regions	9.2	1.1–25.1	.307*

*Significant (*p* < .05)

^a^*β* = standardized regression coefficient with age corrected for sex

**Fig. 2:**
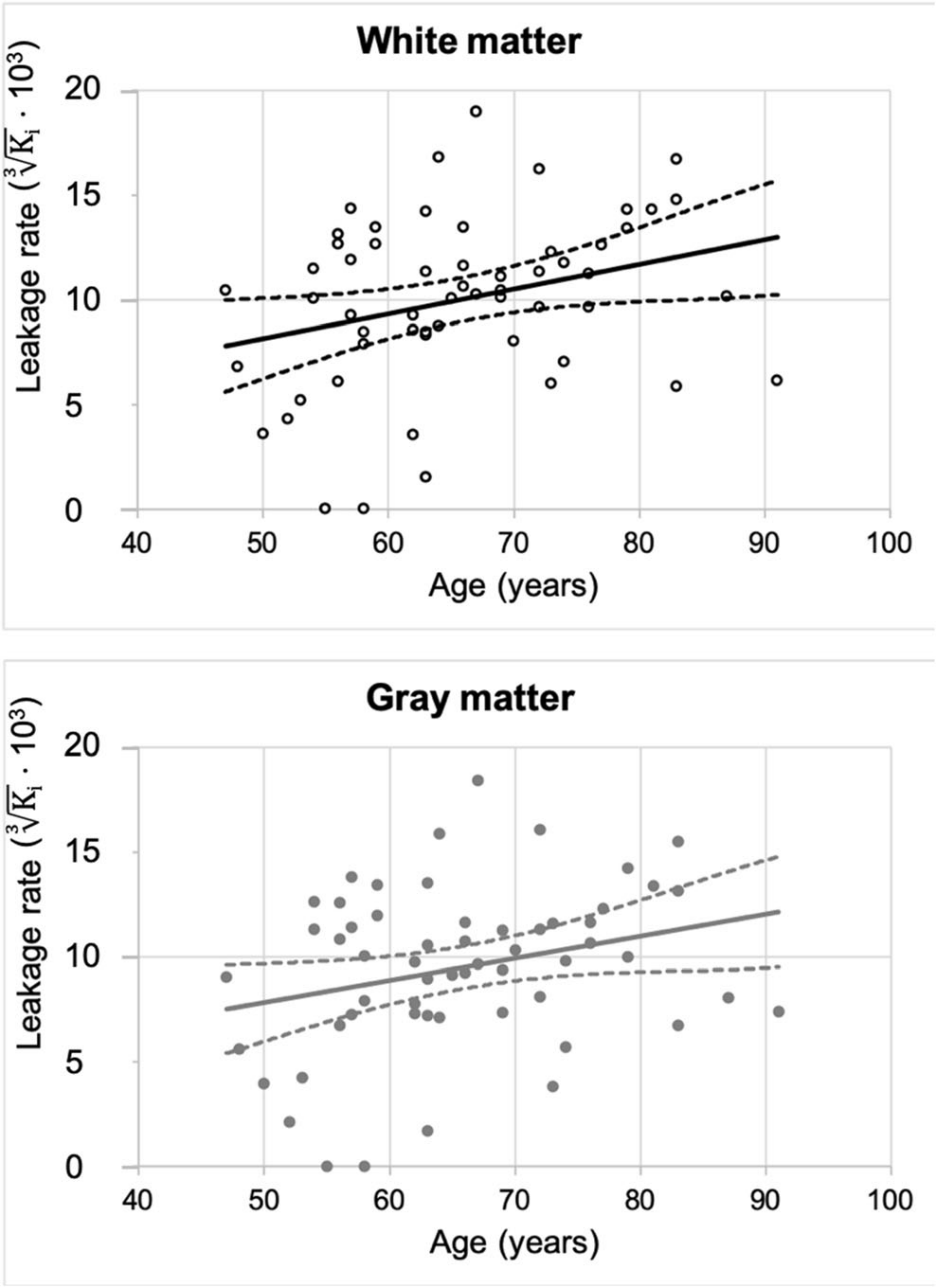
Scatterplots and linear regression of age and leakage rate in the white matter (upper) and gray matter (lower)

*K*_*i*_ was significantly correlated with age, as older participants had significantly more widespread BBB leakage in the cerebral white matter (*β* = .306, *p* = .024) and gray matter (*β* = .286, *p* = .035) (Table 2; Fig. 2).

**Fig. 3:**
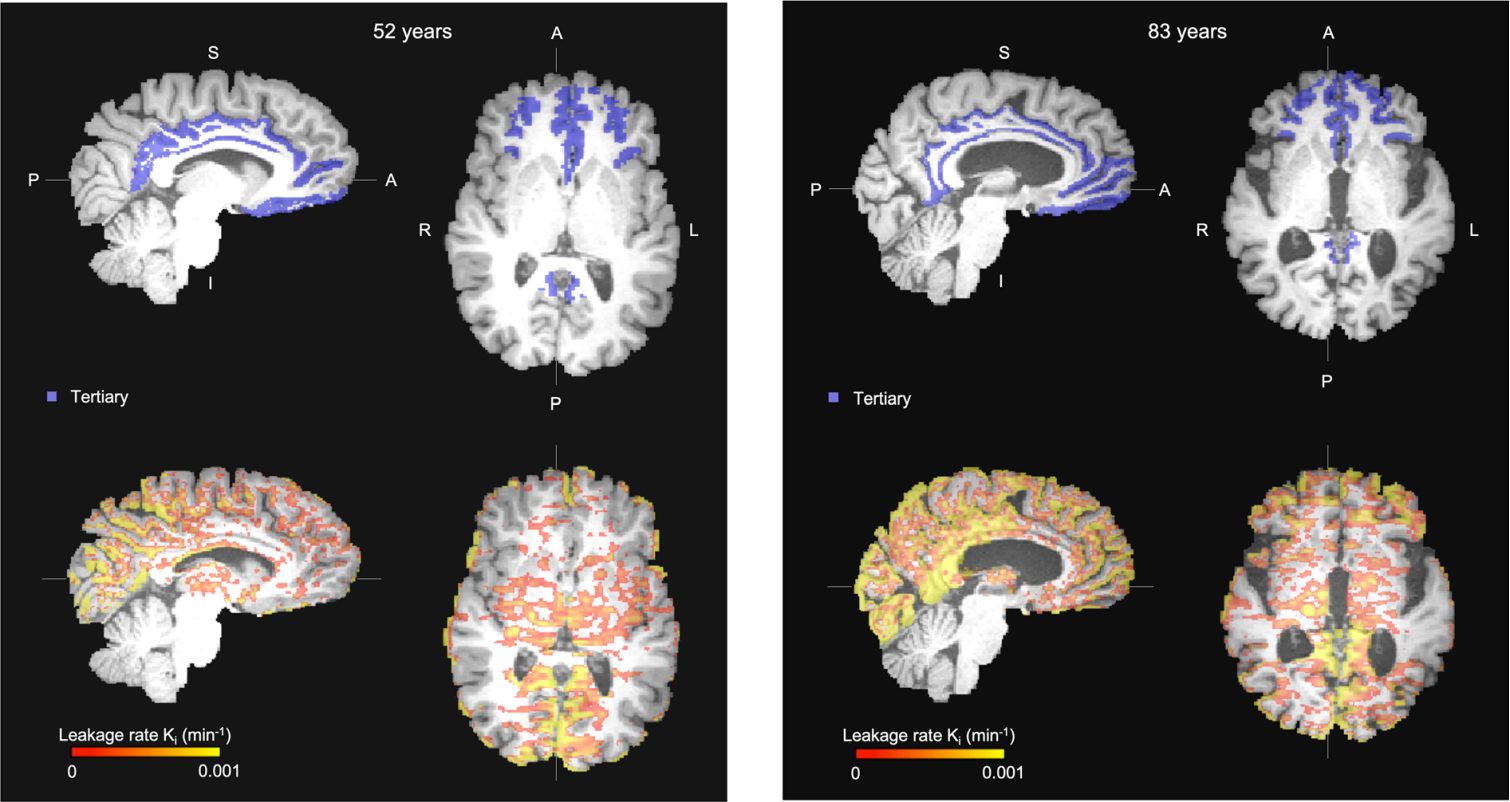
Upper: regions of interest of the tertiary brain regions (in blue), lower: the white and gray matter leakage maps of a younger participant (left; 52 years) and an older participant (right; 83 years). Note the stronger leakage in the older participant, especially in the cingulum

From the three brain region classes, only *K*_*i*_ in the tertiary brain regions was significantly correlated with age (*β* = .301, *p* = .023) (Table 2; Fig. 3).

The associations between *K*_*i*_ and age remained significant when adding the potential confounders mean systolic blood pressure, diabetes, BMI, and smoking habits to the regression analysis.

In the white and gray matter, as well as the tertiary brain regions, significant associations between *K*_*i*_ and age disappeared after correction for WMH volume or cortical thickness. However, significant associations did not depend on hippocampal volume, as correcting for hippocampal volume did not affect the results.

## Discussion

In this study, we hypothesized that BBB disruption would be associated with age in cognitively and neurologically healthy, middle-aged to older individuals. Our results confirmed this hypothesis, as we found higher BBB leakage rate in older individuals from a healthy group when investigating the total cerebral white and gray matter tissue compartments. Also, we found a significant correlation between BBB leakage and age in the tertiary brain regions, but not in the primary or secondary regions, which confirms that BBB disruption increases with age especially in those regions that are known to be most vulnerable to normal age-related deterioration.

Previous studies have demonstrated that decrease in cerebral blood flow and cortical volume reductions are most pronounced in the tertiary brain regions during aging (Fjell et al. 2013; Martin et al. 1991), which supports that age-related detrimental processes especially affect tertiary regions. Our study now shows that BBB disruption could be one of these processes and strongly supports the notion that BBB disruption is an underlying mechanism of normal age-related neurophysiological decline.

Previous studies have already demonstrated that white matter integrity loss and cortical thinning are part of the normal aging process (Maniega et al. 2015; Thambisetty et al. 2010). We now have evidence that BBB disruption increases during normal aging, but this association is largely explained by age-related white matter integrity loss and decrease in cortical thickness. White matter integrity loss, cortical thinning, and BBB disruption all seem to be normal physiological aging phenomena, while hippocampal volume loss is possibly an independent process that might reflect pathology such as found in AD (Wang et al. 2003). For future studies, it would be interesting to also include a measure of regional cerebral blood flow, to investigate how cerebral blood flow reduction correlates with these other age-related processes.

Gaining more knowledge on increase in BBB leakage during normal aging could improve the interpretation and value estimation of leakage rate in pathological conditions. Previous studies have already associated BBB disruption with neurodegenerative disorders, such as AD. It seems reasonable to assume that the distinction between normal and pathological conditions is not a dichotomous, but a gradual phenomenon, and therefore, normal aging individuals most likely still experience some degree of age-related setback. Our finding that BBB leakage is even higher in older adults within the range of normal aging implies that BBB disruption could be an early event in the pathological cascade that in some individuals may eventually be contributing to a neurodegenerative disorder. To gain even better insight into BBB disruption over the age span, studies should be conducted over a broader age range. Including adults around the ages of 20 and 30 years could yield a reference point for BBB leakage at later ages.

Our study uses a cross-sectional design to investigate whether higher leakage rate is associated with older age, so ideally future studies should conduct measurements over time to see whether BBB leakage actually increases as people age. Only longitudinal studies might give information on causality. White matter pathology might damage the vessel walls and generate BBB disruption. However, not only the WMHs but also their nearby normal-appearing white matter has been found to be more permeable, which disputes this notion and could mean that BBB disruption is an initiating factor of the pathological cascade, which triggers white matter pathology (Hainsworth 2019; Riphagen et al. 2018; Wong et al. 2019).

One could also argue on how to define normal aging. Normal aging often means usual aging, where individuals still experience some age-related setback typical for their age group and may have comorbid conditions, as long as these conditions do not significantly affect their functioning (Petersen et al. 1997; Schaie 1994). Our sample for instance had a high mean systolic blood pressure (Table 1). However, these values are not unusual for these age groups (Gaillard et al. 1996) and might be an overestimation, as anxiety while measurements are conducted might elevate blood pressure (Verdecchia et al. 2002). Also, correcting for potential confounders, such as high systolic blood pressure and BMI, did not change our results. Ideally, future studies should also investigate the relation between cardiovascular disease, BBB disruption, and age. For instance, heart failure, which may result in increased central venous pressure, has been shown to cause BBB disruption (Fulop et al. 2019). Moreover, we selected participants from the Maastricht Aging Study, who have participated in this study for 25 years. Participants who are willing and able to participate over such a long period of time are more likely to represent those with an above-average health condition. We were able to find significantly more BBB leakage with older age even within this functionally healthy subsample, and this effect will possibly be even stronger in a more diverse group, so we expect our findings to be relevant for the general aging population.

The integrity of the cerebral vasculature is an important factor in the development of neurodegenerative disorders. Prevention strategies could therefore focus on promoting neurovascular health. Studies are often aimed at the removal of neurotoxic proteins, but also identification of methods to maintain BBB integrity already at an early stage, such as the promising work on restoring NAD^+^ levels, might eventually help increasing the proportion of people who will age in a healthier fashion.

## Conclusion

We found higher BBB leakage rate with older age in healthy individuals, especially in those brain regions most vulnerable to age-related deterioration, which supports the notion that BBB disruption could be an underlying mechanism of normal age-related decline. Promoting neurovascular health and identification of methods to maintain BBB integrity could be a promising avenue to promote healthy aging.

## Data Availability

Data will not be made openly available to secure the privacy of the participants.
